# Identification of a conserved α-helical domain at the N terminus of human DNA methyltransferase 1

**DOI:** 10.1016/j.jbc.2024.105775

**Published:** 2024-02-19

**Authors:** Qi Hu, Maria Victoria Botuyan, Georges Mer

**Affiliations:** 1 Department of Biochemistry and Molecular Biology, Mayo Clinic, Rochester, Minnesota, USA; 2 Department of Cancer Biology, Mayo Clinic, Rochester, Minnesota, USA

**Keywords:** DNMT1, DNA methylation, epigenetics, NMR spectroscopy, small-angle X-ray scattering, new protein domain fold

## Abstract

In vertebrates, DNA methyltransferase 1 (DNMT1) contributes to preserving DNA methylation patterns, ensuring the stability and heritability of epigenetic marks important for gene expression regulation and the maintenance of cellular identity. Previous structural studies have elucidated the catalytic mechanism of DNMT1 and its specific recognition of hemimethylated DNA. Here, using solution nuclear magnetic resonance spectroscopy and small-angle X-ray scattering, we demonstrate that the N-terminal region of human DNMT1, while flexible, encompasses a conserved globular domain with a novel α-helical bundle-like fold. This work expands our understanding of the structure and dynamics of DNMT1 and provides a structural framework for future functional studies in relation with this new domain.

DNA methylation is a major epigenetic modification that regulates chromatin structure and various biological processes in mammals (1, 2, 3, 4). DNA methylation is carried out by four members of the DNA methyltransferase (DNMT) protein family, the best characterized of which is DNMT1. DNMT1 is a 1616-amino acid protein known to encompass a replication foci-targeting sequence (RFTS) domain, two bromo-adjacent-homology domains, and a C-terminal methyltransferase domain (Fig. 1*A*). While absent in lower species, DNMT1 is highly conserved in vertebrates, from *Xenopus laevis* to human.

**Figure 1:**
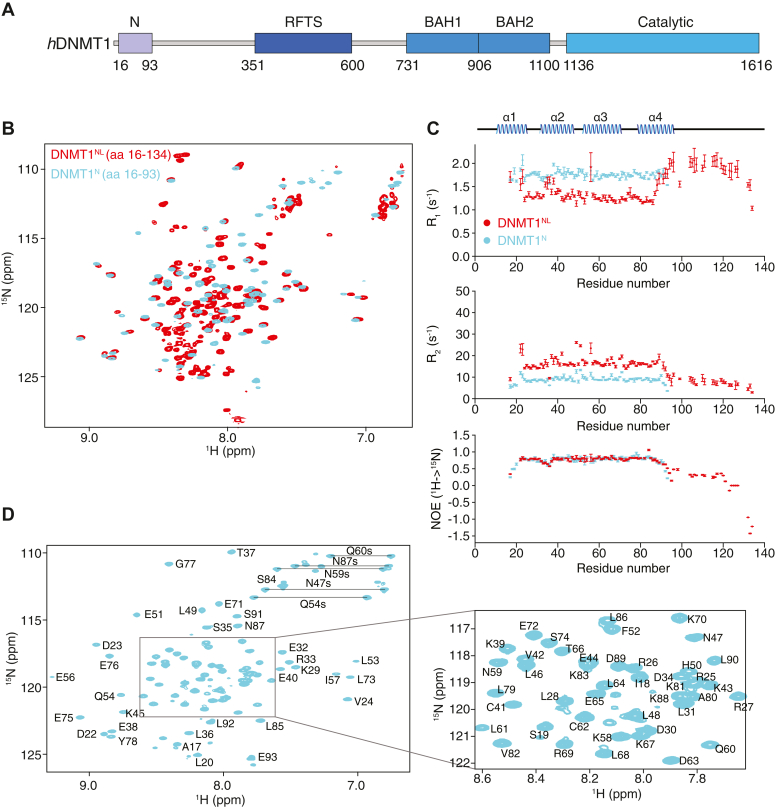
**Identification of a folded segment at the N terminus of DNMT1.***A*, domain structure of human DNMT1 (*h*DNMT1). *B*, overlay of the ^1^H-^15^N HSQC spectra of DNMT1^NL^ (aa 16–134, *red*) and DNMT1^N^ (aa 16–93, *cyan*). *C*, R_1_, R_2_ and ^15^N-{^1^H} NOE for DNMT1^N^ (*cyan*) and DNMT1^NL^ (*red*) are plotted against their residues, with the corresponding secondary structure elements in DNMT1^N^ shown. The R_1_ and R_2_ values were calculated using SPARKY 3.115 with errors determined *via* relaxation curve fitting. For ^15^N-{^1^H} NOEs, shown are the average values ± standard deviation calculated as explained in the Experimental procedures. *D*, ^1^H-^15^N resonance assignment for DNMT1^N^ where side chain signals for asparagine and glutamine residues are indicated by *horizontal lines*. BAH, bromo-adjacent-homology; DNMT1, DNA methyltransferase 1; HSQC, heteronuclear single-quantum coherence; NOE, nuclear overhauser effect; RFTS, replication foci-targeting sequence.

DNA methylation by DNMTs predominantly targets palindromic CpG sites, showing a strong tendency to preferentially methylate CpG sites in a hemimethylated state, although asymmetric methylation at non-CpG sites has also been observed (5). Recent studies have revealed that the establishment and maintenance of DNA methylation involves all DNMTs to varying degrees, in conjunction with DNA demethylases, maintaining a dynamic equilibrium between methylation gain and loss (6). Consequently, knowledge of the DNMT structures is essential for elucidating the specific role played by each member in DNA methylation maintenance.

In the case of DNMT1, many structures containing the RFTS, bromo-adjacent-homology, and catalytic domains have been determined, shedding light on the mechanisms of methylation (7, 8, 9, 10, 11, 12, 13, 14, 15, 16, 17). These studies have deepened our understanding of the modes of action of DNMT1, particularly in relation to pathologic DNMT1 variants implicated in degenerative disorders of the nervous system (18, 19, 20, 21). All structural studies so far have exclusively focused on the segment from residue 350 to the C terminus of DNMT1. The N-terminal region of DNMT1 has received scant attention and has been described as disordered (12), even though limited resistance to proteolysis suggested that it might encompass folded segments (22). Here, using nuclear magnetic resonance (NMR) spectroscopy and small-angle X-ray scattering (SAXS), we identify a hitherto unreported folded domain within the N-terminal region of DNMT1.

## Results

### Identification of a folded domain at the N terminus of DNMT1

We initiated our studies using a recombinant DNMT1 fragment encompassing residues 16 to 134, selected based on predicted secondary structure elements (data not shown), and identified as DNMT1^NL^. The ^1^H-^15^N heteronuclear single-quantum coherence (HSQC) spectrum of DNMT1^NL^ showed well dispersed signals overall, with some variations in signal intensities, indicating that there were structured as well as disordered regions within the protein (Fig. 1*B*). Further inspection of the ^1^H-^15^N relaxation data collected on DNMT1^NL^ revealed that terminal segments comprising residues 16 to 21 and 94 to 134 were intrinsically disordered, with elevated R_1_ and decreased R_2_^15^N relaxation rates and decreased steady-state ^15^N-{^1^H} heteronuclear overhauser effects (NOEs), compared to the rest of the protein (Fig. 1*C*). The rotational correlation time (*τ*_c_) estimated from the average R_1_ and R_2_ values for DNMT1^NL^ was 8.1 ± 2.2 ns, indicating that DNMT1^NL^ is monomeric in solution. By truncating the residues in the C-terminal unstructured region, we produced a shorter version of DNMT1 (residues 16–93), denoted as DNMT1^N^ (Fig. 1*C*). DNMT1^N^ is also a monomer in solution based on its *τ*_c_ value of 5.4 ± 0.6 ns. Compared to the ^1^H-^15^N HSQC of DNMT1^NL^, the spectrum of DNMT1^N^ showed better separation of signals and more homogeneous signal intensities (Fig. 1, *B* and *D*). We therefore used DNMT1^N^ for subsequent structural studies.

The differences in C_α_, C_β_, N, and H_N_ chemical shift values between DNMT1^N^ and DNMT1^NL^ were mostly negligible, except for the C-terminus of DNMT1^N^ near Glu93, as expected, and regions near Ser35 and Leu46-Gln54, where small chemical shift differences were observed in the overlaid ^1^H-^15^N HSQC spectra of DNMT1^N^ and DNMT1^NL^ (Fig. 1*B*). Interestingly, these regions harbor negatively charged residues. The detectable chemical shift perturbations might result from weak transient electrostatic interactions with the extended disordered region of DNMT1^NL^ (aa 94–134).

### Solution NMR structure of DNMT1 N-terminal domain

**Figure 2:**
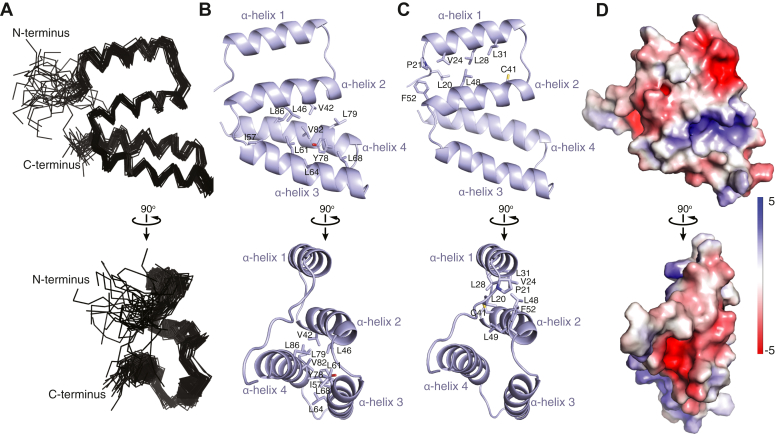
**Three-dimensional structure of DNMT1**^**N**^**.***A*, ensemble of the final 30 lowest-energy NMR structures of DNMT1^N^. *B*, DNMT1^N^ structure in cartoon representation with residues at the interfaces of α-helices 2, 3, and 4 labeled and shown in *stick representation*. *C*, DNMT1^N^ structure in cartoon representation with residues at the interface of α-helices 1 and 2 labeled and shown in *stick representation*. *D*, electrostatic surface potential of DNMT1^N^ calculated using APBS in PyMOL. DNMT1, DNA methyltransferase 1; NMR, nuclear magnetic resonance.

The solution structure of DNMT1^N^ was determined using multidimensional heteronuclear NMR spectroscopy with 937 NOE-based distance restraints, 132 dihedral angle restraints, and 47 ^1^H-^15^N residual dipolar coupling (RDC) restraints for structure calculations (Fig. 2 and Table 1). The NMR structural ensemble of DNMT1^N^ shows four α-helices (Fig. 2*A*). Three of these α-helices—α-helix 2 (aa 38–52), α-helix 3 (aa 55–71), and α-helix 4 (aa 76–91)—constitute a three-helix bundle, with each helix contacting the other two helices (Fig. 2*B*). Additionally, α-helix 2 contacts helix 1 (aa 22–34) (Fig. 2*C*). The plane containing α-helices 1 and 2 is almost perpendicular to the plane formed by α-helices 3 and 4 (Fig. 2, *B* and *C*). The structure of DNMT1^N^ is stabilized by a hydrophobic core formed by the helical bundle and involves Val42 and Leu46 from α-helix 2; Ile57, Leu61, Leu64, and Leu68 from α-helix 3; and Tyr78, Leu79, Val82, and Leu86 from α-helix 4 (Fig. 2*B*). The contact surface between α-helices 1 and 2 is hydrophobic and involves Val24, Leu28, and Leu31 from α-helix 1 and Cys41, Leu48, Leu49, and Phe52 from α-helix 2 (Fig. 2*C*). Furthermore, Leu20, Pro21, Val24, Leu49, and Phe52 form another, smaller, hydrophobic cluster (Fig. 2*C*). Despite numerous hydrophobic contacts, all amide proton signals disappeared within 2 h in an NMR spectroscopy-monitored hydrogen-deuterium exchange experiment (data not shown). This observation suggests a low thermodynamic stability for the domain, related to a small unfolding free energy from the native state to the transient fully unfolded state (23). The electrostatic surface potential of DNMT1^N^ (Fig. 2*D*) does not reveal any remarkable features that could offer clues to the function of this domain.

**Table 1:** NMR and refinement statistics for DNMT1 (residues 16–93)

NMR distance and dihedral constraints	
Distance constraints	
Total NOE	937
Intraresidue	250
Interresidue	688
Sequential (|*i* – *j*| = 1)	288
Medium range (|*i* – *j*| < 5)	305
Long range (|*i* – *j*| > 4)	94
Intermolecular	
Total dihedral angle restraints	132
φ	66
ψ	66
Total RDC restraints	47
Q factor	0.16
Structure statistics	
Violations (mean and s.d.)	
Distance constraints (Å)	0.032 ± 0.002
Dihedral angle constraints (°)	0.132 ± 0.017
Max. dihedral angle violation (°)	4.613
Max. distance constraint violation (Å)	0.470
Deviations from idealized geometry	
Bond lengths (Å)	0.005 ± 0.000
Bond angles (°)	0.665 ± 0.008
Impropers (°)	0.516 ± 0.015
Average pairwise r.m.s. deviation^a^ (Å)	
Heavy	1.51 ± 0.21
Backbone	0.70 ± 0.24
Average r.m.s. deviation to mean structure^a^ (Å)	
Heavy	1.06 ± 0.13
Backbone	0.49 ± 0.18
Ramachandran plot summary from Procheck (%)	
Most favored regions	96.4
Additionally allowed regions	2.4
Generously allowed regions	1.1
Disallowed regions	0.1

a DNMT1 (residues 22–90).

**Figure 3:**
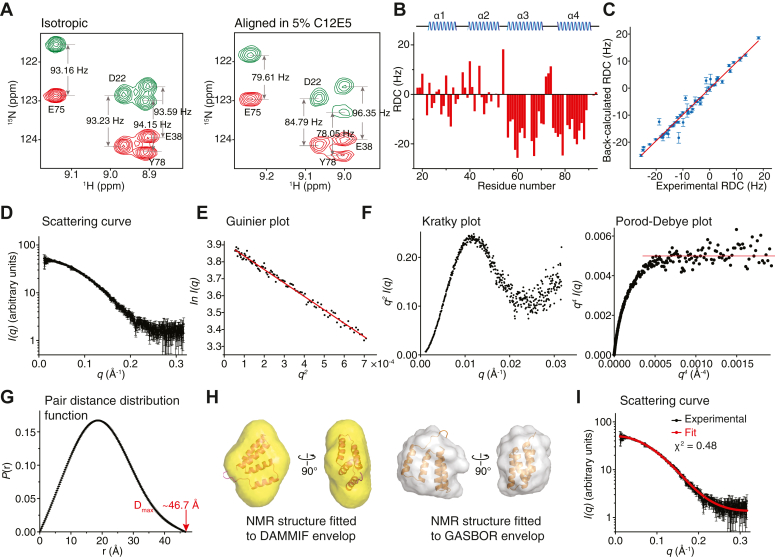
**Validation of the DNMT1**^**N**^**structure.***A*, representative 2D IPAP ^1^H-^15^N-HSQC spectra for DNMT1^N^ from which RDCs were measured. *Left*, spectrum of isotropic DNMT1^N^ sample. *Right*, spectrum of DNMT1^N^ aligned in 5% C12E5/95% n-hexanol. *B*, experimentally measured backbone ^1^H-^15^N RDCs for DNMT1^N^. Secondary structure elements are shown on the *top*. *C*, comparison between experimental and back-calculated ^1^H-^15^N RDCs. For each back-calculated RDC, shown is the mean value ± standard deviation calculated from the 30 NMR structures. *D*, SAXS scattering data of DNMT1^N^. *E*, Guinier plot at low angles (*q∗R*_*g*_ <1.3) where *R*_g_ is the radius of gyration. *F*, Kratky plot (*left*) and Porod-Debye plot (*right*) showing a linear plateau (*red line*) agreeing with a globular protein with limited flexibility. *G*, pair distance distribution function showing a D_max_ of ∼46.7 Å. *H*, *left*, DNMT1^N^ NMR structure rigid-body-docked to the *ab initio* molecular envelope of DNMT1^N^ generated using DAMMIF. *Right*, DNMT1^N^ NMR structure docked to GASBOR bead model of DNMT1^N^. *I*, SAXS scattering curve back-calculated from the lowest energy NMR structure (*red*) overlaid to experimental scattering data (*black*). Goodness of fit χ^2^ is indicated. DNMT1, DNA methyltransferase 1; HSQC, heteronuclear single-quantum coherence; NMR, nuclear magnetic resonance; RDC, residual dipolar coupling; SAXS, small-angle X-ray scattering.

The RDC restrains were instrumental in refining and validating the relative orientations of the different regions of DNMT1^N^. Residues 55 to 90, which cover α-helices 3 and 4 in the structure, have uniformly negative RDC values except for the residues connecting these α-helices (Fig. 3, *A* and *B*). The RDC values for residues 16 to 54, corresponding to α-helices 1 and 2, are less uniform, indicating that α-helices 1 and 2 have different orientations from those of α-helices 3 and 4. There is excellent agreement between the experimentally measured and back-calculated RDCs (Fig. 3*C*).

### SAXS analysis of DNMT1 N-terminal domain

We used SAXS (24, 25) to examine the global fold and oligomerization state of DNMT1^N^ and thereby further evaluate the NMR-derived structure (Fig. 3*D* and Table S1). The SAXS Guinier plot (26) of DNMT1^N^ was characteristic of a homogeneous sample (Fig. 3*E*). Furthermore, the Kratky and Porod-Debye plots (27) showed that the protein was globular with limited flexibility in the N and C termini (Fig. 3*F*). We derived a radius of gyration of ∼15.4 Å and a maximum dimension D_max_ of ∼46.7 Å for DNMT1^N^, consistent with a monomeric state (Fig. 3*G*). The overall shape of DNMT1^N^ was calculated by *ab initio* model reconstruction using GASBOR and DAMMIF from the ATSAS SAXS data analysis software package (28). GASBOR reconstructs the protein structure by a chain-like ensemble of dummy residues while DAMMIF does the reconstruction through assembly of densely packed spheres. Superposition of the NMR structure with the envelopes generated from DAMMIF and GASBOR showed good fit to both envelopes (Fig. 3*H*). Further evaluation using FoXS (29) demonstrated high consistency of the SAXS data with the DNMT1^N^ NMR ensemble (Fig. 3*I*). The SAXS and NMR approaches indicate that DNMT1^N^ is a monomer in solution.

### DNMT1^N^ adopts a novel fold

**Figure 4:**
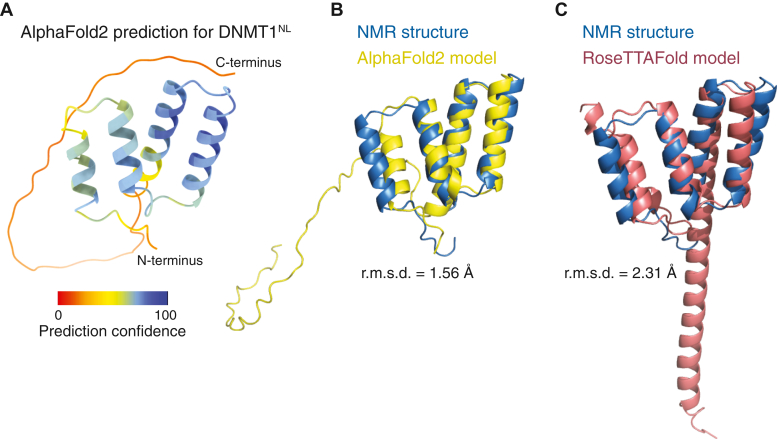
**DNMT1 structure prediction using AlphaFold2 and RoseTTAFold.***A*, cartoon representation of AlphaFold2-predicted human DNMT1^NL^ structure color coded according to the per-residue confidence metric pLDDT. *B* and *C*, NMR structure of DNMT1^N^ overlaid to models generated using AlphaFold2 (*B*) and RoseTTAFold (*C*). The r.m.s.d. values calculated for the backbone Cα, C, and N atoms of residues Glu22 to Leu90 are indicated. DNMT1, DNA methyltransferase 1; NMR, nuclear magnetic resonance.

A search using the DALI server against the Protein Data Bank and AlphaFold-predicted human proteome (30, 31, 32) identified an N-terminal motif in human DNMT1 that closely matches our DNMT1^N^ NMR structure (Fig. 4*A*). The root mean square deviation (r.m.s.d.) between the lowest energy NMR structure and the predicted model was 1.56 Å for the backbone C_α_, C, and N atoms of residues Glu22 to Leu90 (Fig. 4*B*). No other predicted protein structures exhibited a similar arrangement of four α-helices. Therefore, we conclude that DNMT1^N^ adopts a novel fold. No other secondary structure elements were predicted beyond DNMT1^N^ and before the RFTS domain. RoseTTAFold (33) also produced a structure comparable to that of DNMT1^N^ (r.m.s.d. = 2.31 Å for the backbone C_α_, C, and N atoms of residues Glu22 to Leu90), but with a 33-residue C-terminal helical extension to the fourth α-helix, not present in the experimental structure (Fig. 4*C*).

## Discussion

We discovered a new folded domain of unknown function at the N terminus of DNMT1 (DNMT1^N^). Due to the chromatin association properties of DNMT1 (34), we investigated the binding of DNMT1^NL^ to the nucleosome core particle, but no interaction was detected (data not shown). However, there are clues that DNMT1^N^ has regulatory roles. Notably, it has been shown that through alternative RNA splicing of a sex-specific exon, DNMT1 from mammalian oocytes lacks a segment that matches DNMT1^N^ and is sequestered in the cytoplasm (35). Moreover, there is evidence that DNMT1^N^ interacts with the E-cadherin transcriptional repressor SNAIL1, with speculation that DNMT1 promotes gene expression by impeding the interaction of SNAIL1 with the E-cadherin promoter (36, 37). It has also been reported that DNMT1^N^ interacts with DMAP1, a protein that preferentially activates DNMT1-mediated DNA methylation at sites of homologous recombination repair in response to DNA double-strand breaks (38, 39). In addition, deletion of DNMT1^N^ in breast cancer cell lines was shown to diminish the histone deacetylase inhibitor LBH589-induced ubiquitylation-dependent degradation of DNMT1 and resulted in genomic hypermethylation (40). Consistently, an isoform of DNMT1 that lacks the N-terminal domain exhibited higher stability than full-length DNMT1 *in vivo* (41, 42). The underlying mechanism is unclear but likely involves cross-talks among several different post-translational modifications on DNMT1, such as methylation and acetylation. Intriguingly, Lys70 in DNMT1^N^ was found to be methylated by protein methyltransferase G9a (43, 44). Whether or not this modification contributes to the regulation of DNMT1 level in cells has not been investigated.

In conclusion, because the structure of DNMT1^N^ represents a novel fold, it cannot be used to suggest a possible function. However, based on what has been published so far, we can speculate that this helical domain is a protein-interaction module. The structure of DNMT1^N^ will be helpful for the rational design of single-point mutations aimed at deciphering the function of this domain using cell biology approaches.

## Experimental procedures

### Protein expression and purification

The N-terminal domain of human DNMT1 (residues 16–134), denoted as DNMT1^NL^, was cloned with a tobacco etch virus protease cleavable N-terminal His_6_-tag in a pET15b-derived expression system. A shorter version (residues 16–93), denoted as DNMT1^N^, was made by inserting a stop codon (TAA) after Glu93. All proteins were produced in BL21(DE3) *E. coli* cells grown in M9 media prepared with ^15^N-labeled NH_4_Cl and unlabeled or ^13^C-enriched glucose. The cells were initially grown at 37 °C to an *A*_600_ of ∼0.5, then at 15 °C to an *A*_600_ of ∼0.6 before being induced with 1 mM isopropyl β-D-1-thiogalactopyranoside for 16 h. The harvested cells were lysed using an EmulsiFlex C5 homogenizer (Avestin). The proteins were initially purified by Ni^2+^-nitrilotriacetic acid agarose chelation chromatography (QIAGEN) using buffers of 50 mM sodium phosphate, pH 7.5, 300 mM NaCl with 5-, 20- and 200-mM imidazole for the binding, washing, and elution steps, respectively. The His_6_-tags were cleaved by overnight incubation with tobacco etch virus protease at 4 °C. The proteins were further purified by size-exclusion chromatography using a HiLoad 16/60 Superdex 75 column (Cytiva) and a running buffer of 50 mM sodium phosphate, pH 7.5, 300 mM NaCl.

### NMR spectroscopy

All NMR experiments were performed at 25 °C using a Bruker Avance III 700 MHz spectrometer equipped with a triple-resonance cryoprobe. The NMR buffer for the ^15^N- and ^15^N-/^13^C-labeled DNMT1^N^ and DNMT1^NL^ protein samples was 20 mM MES/Bis-Tris, 50 mM NaCl, pH 6.0. The NMR spectra were processed with NMRPipe (45) and analyzed using SPARKY 3.115 (T. D. Goddard and D. G. Kneller, SPARKY 3, University of California, San Francisco). For resonance assignments, ^13^C,^15^N-labeled DNMT1^N^ and DNMT1^NL^ were used to collect a series of standard triple-resonance spectra including HNCO, HN(CA)CO, HNCA, HN(CO)CA, HNCACB, CBCA(CO)NH, HCCH-COSY, HCCH-TOCSY, and HBHA(CO)NH (46, 47, 48). We were able to assign 96.6% of the backbone and 78.3% of the sidechain carbon, proton, and nitrogen resonances.

The ^15^N NMR relaxation studies were carried out on both ^15^N-labeled DNMT1^N^ and DNMT1^NL^. Longitudinal (R_1_) and transverse (R_2_) relaxation rates for backbone ^1^H-^15^N and ^15^N-{^1^H} steady-state NOEs were measured on these samples and analyzed using established methods (49, 50, 51). Ten relaxation delays (100, 300, 500, 600, 800, 1000, 1200, 1500, 1600, and 2000 ms) were used for R_1_, while 11 (4, 8, 16, 20, 28, 32, 40, 60, 80, 100, and 200 ms) were used for R_2_. The ^15^N-{1H} NOE ratios were obtained from a reference experiment without proton irradiation and a steady-state experiment with proton irradiation for 3 s. The standard deviations of ^15^N-{^1^H} NOEs were calculated based on the measured background noise levels, as previously reported (49), using Equation 1:

(1) $$\frac{\sigma_{\text{NOE}}}{\text{NOE}}={\left({\left(\frac{\sigma_{\text{Isat}}}{I_{\text{sat}}}\right)}^{2}+{\left(\frac{\sigma_{\text{Iunsat}}}{I_{\text{unsat}}}\right)}^{2}\right)}^{\frac{1}{2}}$$

where I_sat_ and I_unsat_ are the measured intensities of the resonances in the presence and absence of proton saturation, respectively. σ_Isat_ and σ_Iunsat_ are the standard deviations of the noise in the spectra.

For structure determination of DNMT1^N^, distance restraints were obtained from the analysis of 3D ^15^N-edited NOESY HSQC spectra collected in 90%/10% H_2_O/D_2_O and ^13^C-edited NOESY HSQC spectra collected in 90%/10% H_2_O/D_2_O and in 100% D_2_O. The mixing time for these experiments was 160 ms. In total, 937 NOE-based distance restraints were used and categorized into seven bins with upper limits of 3.0, 3.5, 4.0, 4.5, 5.0, 5.5, and 6.0 Å. Also included in the structure calculations were 132 backbone dihedral angle φ and ψ restraints derived from the analysis of H_α_, H_N_, ^13^C_α_, ^13^C_β_, ^13^C, and ^15^N chemical shifts using TALOS+ (52) and 47 ^1^H-^15^N RDC restraints out of 60 that were measured. The RDCs were measured in a 5% pentaethylene glycol monododecyl ether (C12E5)/95% n-hexanol mixture using 2D ^1^H-^15^N IPAP HSQC experiments (53, 54, 55). The RDC alignment tensor magnitude Da and rhombicity used in the structure calculations were −11.00 and 0.61, respectively. The structures were calculated and refined using XPLOR-NIH by employing a simulated annealing protocol for torsion angle dynamics (56, 57). A total of 200 structures were initially calculated, from which 30 structures with the lowest energies were used for further refinement.

A total of 60 measured RDCs, 47 of which were used for structure calculations, were compared to RDCs back-calculated from the 30 NMR structures using PALES (58), giving a Pearson’s linear correlation coefficient of 0.97. The quality factor *Q* factor (59), which evaluates the agreement between the RDCs back-calculated from the structures and the observed RDCs, was used as a figure of merit for the goodness of fit of the calculated structures to the experimental data. In Equation 2, *Q* is the quality factor; *RMS* stands for root mean square; *D*_calc_ and *D*_obs_ are the back-calculated and measured residual dipolar couplings, respectively.

(2) $$Q=\frac{RMS\;\left(D_{\text{calc}}-D_{\text{obs}}\;\right)}{RMS\;\left(D_{\text{obs}}\right)}$$

All molecular representations were generated using PyMOL (The PyMOL Molecular Graphics System, Schrödinger, LLC — https://pymol.org/2/). The electrostatic potential was calculated using APBS (60).

### Small-angle X-ray scattering

The SAXS data were collected at the SIBYLS beamline 12.3.1, Advanced Light Source, Lawrence Berkeley National Laboratory, on several DNMT1^N^ samples (Table S1). For each sample, the scattering intensities were measured at three different protein concentrations (1, 2, and 3 mg/ml), demonstrating the absence of concentration dependence. Three different exposure times of 0.5, 1.0, and 5.0 s were used for each sample, and data were monitored for radiation damage-dependent aggregation. Scattering data were plotted as a function of *q* = 4π[sin(θ/2)]/λ, where θ is the scattering angle and λ is the X-ray wavelength, subtracting for each curve the scattering data collected for just the buffer alone. The curves were rescaled for the solute concentrations and extrapolated to infinite dilution. All data analyses were performed using PRIMUS, version 3.0, from ATSAS 2.4.2 (28). GNOM was used to generate the pair distance distribution function (*P*(r)) from which the maximum particle dimension (D_max_) was estimated. The radius of gyration (*R*_g_) was estimated using the Guinier plot (61). Divergent low-*q* data points exhibiting artifacts from beam-stopper scattering and data points of *q* >0.25 Å^−1^ were not included in Guinier and *P*(r) analysis. The output of GNOM was used as input for DAMMIF to calculate the overall shape of DNMT1^N^. Twenty independent runs were conducted, and the generated models were averaged using DAMAVER to build a consensus molecular envelope. An *ab initio* envelope was also created using GASBOR as a comparison. SUPCOMB was used to superimpose the *ab initio* envelopes and NMR structures. The various software, including PRIMUS, GNOM, DAMMIF, DAMAVER, GASBOR, and SUPCOMB, were all from the ATSAS 2.4.2 program package (28).

### AlphaFold2 and RoseTTAFold predictions

The DNMT1^NL^ 3D structure predictions were performed using the AlphaFold2 Colab server (https://colab.research.google.com/github/sokrypton/ColabFold/blob/main/AlphaFold2.ipynb) and RoseTTAFold server (robetta.bakerlab.org).

## Data availability

Coordinates for the NMR ensemble have been deposited in the Protein Data Bank with accession number 8V9U. NMR chemical shift assignments have been deposited in the Biological Magnetic Resonance Data Bank with accession number 31134.

## Supporting information

This article contains supporting information.

## Conflict of interest

The authors declare that they have no conflicts of interest with the contents of this article.

## References

[1] Edwards J.R., Yarychkivska O., Boulard M., Bestor T.H. (2017). DNA methylation and DNA methyltransferases. Epigenet. Chromatin.

[2] Gowher H., Jeltsch A. (2018). Mammalian DNA methyltransferases: new discoveries and open questions. Biochem. Soc. Trans..

[3] Kim M., Costello J. (2017). DNA methylation: an epigenetic mark of cellular memory. Exp. Mol. Med..

[4] Schubeler D. (2015). Function and information content of DNA methylation. Nature.

[5] He Y., Ecker J.R. (2015). Non-CG methylation in the human genome. Annu. Rev. Genomics Hum. Genet..

[6] Jeltsch A., Jurkowska R.Z. (2014). New concepts in DNA methylation. Trends Biochem. Sci..

[7] Takeshita K., Suetake I., Yamashita E., Suga M., Narita H., Nakagawa A. (2011). Structural insight into maintenance methylation by mouse DNA methyltransferase 1 (Dnmt1). Proc. Natl. Acad. Sci. U. S. A..

[8] Song J., Rechkoblit O., Bestor T.H., Patel D.J. (2011). Structure of DNMT1-DNA complex reveals a role for autoinhibition in maintenance DNA methylation. Science.

[9] Syeda F., Fagan R.L., Wean M., Avvakumov G.V., Walker J.R., Xue S. (2011). The replication focus targeting sequence (RFTS) domain is a DNA-competitive inhibitor of Dnmt1. J. Biol. Chem..

[10] Zhang Z.M., Liu S., Lin K., Luo Y., Perry J.J., Wang Y. (2015). Crystal structure of human DNA methyltransferase 1. J. Mol. Biol..

[11] Kanada K., Takeshita K., Suetake I., Tajima S., Nakagawa A. (2017). Conserved threonine 1505 in the catalytic domain stabilizes mouse DNA methyltransferase 1. J. Biochem..

[12] Ishiyama S., Nishiyama A., Saeki Y., Moritsugu K., Morimoto D., Yamaguchi L. (2017). Structure of the Dnmt1 reader module complexed with a unique two-mono-ubiquitin mark on histone H3 reveals the basis for DNA methylation maintenance. Mol. Cell.

[13] Ye F., Kong X., Zhang H., Liu Y., Shao Z., Jin J. (2018). Biochemical studies and molecular dynamic simulations reveal the molecular basis of conformational changes in DNA methyltransferase-1. ACS Chem. Biol..

[14] Li T., Wang L., Du Y., Xie S., Yang X., Lian F. (2018). Structural and mechanistic insights into UHRF1-mediated DNMT1 activation in the maintenance DNA methylation. Nucleic Acids Res..

[15] Ren W., Fan H., Grimm S.A., Guo Y., Kim J.J., Yin J. (2020). Direct readout of heterochromatic H3K9me3 regulates DNMT1-mediated maintenance DNA methylation. Proc. Natl. Acad. Sci. U. S. A..

[16] Ren W., Fan H., Grimm S.A., Kim J.J., Li L., Guo Y. (2021). DNMT1 reads heterochromatic H4K20me3 to reinforce LINE-1 DNA methylation. Nat. Commun..

[17] Kikuchi A., Onoda H., Yamaguchi K., Kori S., Matsuzawa S., Chiba Y. (2022). Structural basis for activation of DNMT1. Nat. Commun..

[18] Klein C.J., Botuyan M.V., Wu Y., Ward C.J., Nicholson G.A., Hammans S. (2011). Mutations in DNMT1 cause hereditary sensory neuropathy with dementia and hearing loss. Nat. Genet..

[19] Winkelmann J., Lin L., Schormair B., Kornum B.R., Faraco J., Plazzi G. (2012). Mutations in DNMT1 cause autosomal dominant cerebellar ataxia, deafness and narcolepsy. Hum. Mol. Genet..

[20] Klein C.J., Bird T., Ertekin-Taner N., Lincoln S., Hjorth R., Wu Y. (2013). DNMT1 mutation hot spot causes varied phenotypes of HSAN1 with dementia and hearing loss. Neurology.

[21] Baets J., Duan X., Wu Y., Smith G., Seeley W.W., Mademan I. (2015). Defects of mutant DNMT1 are linked to a spectrum of neurological disorders. Brain.

[22] Suetake I., Hayata D., Tajima S. (2006). The amino-terminus of mouse DNA methyltransferase 1 forms an independent domain and binds to DNA with the sequence involving PCNA binding motif. J. Biochem..

[23] Bai Y., Sosnick T.R., Mayne L., Englander S.W. (1995). Protein folding intermediates: native-state hydrogen exchange. Science.

[24] Korasick D.A., Tanner J.J. (2018). Determination of protein oligomeric structure from small-angle X-ray scattering. Protein Sci..

[25] Grawert T.W., Svergun D.I. (2020). Structural modeling using solution small-angle X-ray scattering (SAXS). J. Mol. Biol..

[26] Putnam C.D. (2016). Guinier peak analysis for visual and automated inspection of small-angle X-ray scattering data. J. Appl. Crystallogr..

[27] Rambo R.P., Tainer J.A. (2011). Characterizing flexible and intrinsically unstructured biological macromolecules by SAS using the Porod-Debye law. Biopolymers.

[28] Petoukhov M.V., Franke D., Shkumatov A.V., Tria G., Kikhney A.G., Gajda M. (2012). New developments in the ATSAS program package for small-angle scattering data analysis. J. Appl. Cryst..

[29] Schneidman-Duhovny D., Hammel M., Sali A. (2010). FoXS: a web server for rapid computation and fitting of SAXS profiles. Nucleic Acids Res..

[30] Holm L., Sander C. (1995). Dali: a network tool for protein structure comparison. Trends Biochem. Sci..

[31] Holm L., Laiho A., Toronen P., Salgado M. (2023). DALI shines a light on remote homologs: one hundred discoveries. Protein Sci..

[32] Jumper J., Evans R., Pritzel A., Green T., Figurnov M., Ronneberger O. (2021). Highly accurate protein structure prediction with AlphaFold. Nature.

[33] Baek M., DiMaio F., Anishchenko I., Dauparas J., Ovchinnikov S., Lee G.R. (2021). Accurate prediction of protein structures and interactions using a three-track neural network. Science.

[34] Schrader A., Gross T., Thalhammer V., Langst G. (2015). Characterization of Dnmt1 binding and DNA methylation on nucleosomes and nucleosomal arrays. PLoS One.

[35] Mertineit C., Yoder J.A., Taketo T., Laird D.W., Trasler J.M., Bestor T.H. (1998). Sex-specific exons control DNA methyltransferase in mammalian germ cells. Development.

[36] Espada J., Peinado H., Lopez-Serra L., Setien F., Lopez-Serra P., Portela A. (2011). Regulation of SNAIL1 and E-cadherin function by DNMT1 in a DNA methylation-independent context. Nucleic Acids Res..

[37] Espada J. (2012). Non-catalytic functions of DNMT1. Epigenetics.

[38] Rountree M.R., Bachman K.E., Baylin S.B. (2000). DNMT1 binds HDAC2 and a new co-repressor, DMAP1, to form a complex at replication foci. Nat. Genet..

[39] Lee G.E., Kim J.H., Taylor M., Muller M.T. (2010). DNA methyltransferase 1-associated protein (DMAP1) is a co-repressor that stimulates DNA methylation globally and locally at sites of double strand break repair. J. Biol. Chem..

[40] Zhou Q., Agoston A.T., Atadja P., Nelson W.G., Davidson N.E. (2008). Inhibition of histone deacetylases promotes ubiquitin-dependent proteasomal degradation of DNA methyltransferase 1 in human breast cancer cells. Mol. Cancer Res..

[41] Ding F., Chaillet J.R. (2002). *In vivo* stabilization of the Dnmt1 (cytosine-5)- methyltransferase protein. Proc. Natl. Acad. Sci. U. S. A..

[42] Agoston A.T., Argani P., Yegnasubramanian S., De Marzo A.M., Ansari-Lari M.A., Hicks J.L. (2005). Increased protein stability causes DNA methyltransferase 1 dysregulation in breast cancer. J. Biol. Chem..

[43] Chang Y., Sun L., Kokura K., Horton J.R., Fukuda M., Espejo A. (2011). MPP8 mediates the interactions between DNA methyltransferase Dnmt3a and H3K9 methyltransferase GLP/G9a. Nat. Commun..

[44] Rathert P., Dhayalan A., Murakami M., Zhang X., Tamas R., Jurkowska R. (2008). Protein lysine methyltransferase G9a acts on non-histone targets. Nat. Chem. Biol..

[45] Delaglio F., Grzesiek S., Vuister G.W., Zhu G., Pfeifer J., Bax A. (1995). NMRPipe: a multidimensional spectral processing system based on UNIX pipes. J. Biomol. NMR.

[46] Ferentz A.E., Wagner G. (2000). NMR spectroscopy: a multifaceted approach to macromolecular structure. Q. Rev. Biophys..

[47] Mer G., Bochkarev A., Gupta R., Bochkareva E., Frappier L., Ingles C.J. (2000). Structural basis for the recognition of DNA repair proteins UNG2, XPA, and RAD52 by replication factor RPA. Cell.

[48] Botuyan M.V., Mer G., Yi G.S., Koth C.M., Case D.A., Edwards A.M. (2001). Solution structure and dynamics of yeast elongin C in complex with a von Hippel-Lindau peptide. J. Mol. Biol..

[49] Farrow N.A., Muhandiram R., Singer A.U., Pascal S.M., Kay C.M., Gish G. (1994). Backbone dynamics of a free and phosphopeptide-complexed Src homology 2 domain studied by ^15^N NMR relaxation. Biochemistry.

[50] Dayie K.T., Wagner G., Lefèvre J.F. (1996). Theory and practice of nuclear spin relaxation in proteins. Annu. Rev. Phys. Chem..

[51] Mer G., Dejaegere A., Stote R., Kieffer B., Lefèvre J.-F. (1996). Structural dynamics of PMP-D2: an experimental and theoretical study. J. Phys. Chem..

[52] Shen Y., Delaglio F., Cornilescu G., Bax A. (2009). TALOS+: a hybrid method for predicting protein backbone torsion angles from NMR chemical shifts. J. Biomol. NMR.

[53] Tjandra N., Bax A. (1997). Direct measurement of distances and angles in biomolecules by NMR in a dilute liquid crystalline medium. Science.

[54] Rückert M., Otting G. (2000). Alignment of biological macromolecules in novel nonionic liquid crystalline media for NMR experiments. J. Am. Chem. Soc..

[55] Yao L., Ying J., Bax A. (2009). Improved accuracy of ^15^N-^1^H scalar and residual dipolar couplings from gradient-enhanced IPAP-HSQC experiments on protonated proteins. J. Biomol. NMR.

[56] Schwieters C.D., Kuszewski J.J., Tjandra N., Clore G.M. (2003). The Xplor-NIH NMR molecular structure determination package. J. Magn. Reson..

[57] Schwieters C.D., Kuszewski J.J., Clore G.M. (2006). Using Xplor-NIH for NMR molecular structure determination. Progr. NMR Spec..

[58] Zweckstetter M. (2008). NMR: prediction of molecular alignment from structure using the PALES software. Nat. Protoc..

[59] Cornilescu G., Marquardt J.L., Ottiger M., Bax A. (1998). Validation of protein structure from anisotropic carbonyl chemical shifts in a dilute liquid crystalline phase. J. Am. Chem. Soc..

[60] Jurrus E., Engel D., Star K., Monson K., Brandi J., Felberg L.E. (2018). Improvements to the APBS biomolecular solvation software suite. Protein Sci..

[61] Guinier A., Fournet G. (1955). Small-Angle Scattering of X-Rays.

