# Supplementary material for: Identification of a conserved α-helical domain at the N terminus of human DNA methyltransferase 1
Source: J Biol Chem. 2024 Feb 19;300(3):105775. doi: 10.1016/j.jbc.2024.105775 (PMC10950863; doi:10.1016/j.jbc.2024.105775)
Supplement: Table S1 [file mmc1.pdf]

Table S1 SAXS reporting data

**Sample details**	
	DNMT1^N
Organism	*Homo Sapiens*
Source	*E. coli*
Description: sequence & ligands	P26358 DNMT1 (residues 16-93)
Extinction coefficient ε (wavelength and units)	280 nm, 1,490 M^{-1} cm^{-1}
Molecular mass (Da)	9,152.533
Concentration (range/values) measured	1, 2, 3 mg/mL
Solvent composition	25 mM sodium phosphate, pH 6.9, 50 mM NaCl
**SAXS data collection parameters**	
Source	SIBYLS beamline 12.3.1 at the Advanced Light Source
Wavelength (Å)	1.127
Beam geometry (size, sample-to-detector distance)	0.1 x 0.1 mm, 1.5 m
q-measurement range ($Å^{-1}$)	0.011-0.316
Method for monitoring radiation damage	Varied exposure lengths were compared against one another to assess damage
Exposure time, number of exposures	0.5 s, 1 s, 6 s exposures for each concentration
Sample configuration	A 1 mm sample was placed between two mica windows in a transmission geometry
Sample temperature	10 ºC
**Software employed for SAXS data reduction, analysis and interpretation**	
SAS data reduction	Subtraction using established programs at SIBYLS beamline 12.3.1
Basic analyses and merging of curves.	PRIMUS from ATSAS 2.4.2
Shape/bead modeling	GASBOR 2.3i, DAMMIF
**Structural parameters**	
Guinier analysis	
	DNMT1^N
$I(0)$ (in detector units)	50.10 ± 0.26
R_g (Å)	15.40 ± 0.13
q-range ($Å^{-1}$)	0.011-0.316
qR_g max	1.3
$P(r)$ analysis	
	DNMT1^N
$I(0)$ (in detector units)	49.93± 0.16
R_g (Å)	15.40 ± 0.04
d_{max} (Å)	46.70
q-range ($Å^{-1}$)	0.011-0.316
Porod volume ($Å^3$)	19377
**Shape modelling results**	
	DNMT1^N
GASBOR 2.3i	
q-range for fitting	0.011-0.316
Symmetry	P1
χ^2 value	1.30
d_{max} (Å), R_g (Å)	46.70, 15.40
**Atomistic modelling**	
	DNMT1^N
FoXS	
NMR structure	PDB:8V9U
q-range for fitting	0.011-0.316
Symmetry assumptions	P1
χ^2 value/range	0.48
c_1, c_2	1.01, 1.20
Predicted R_g	14.72
